# An open source algorithm to detect natural gas leaks from mobile methane survey data

**DOI:** 10.1371/journal.pone.0212287

**Published:** 2019-02-13

**Authors:** Zachary D. Weller, Duck Keun Yang, Joseph C. von Fischer

**Affiliations:** 1 Department of Statistics, Colorado State University, Fort Collins, Colorado, United States of America; 2 Department of Computer Science, Colorado State University, Fort Collins, Colorado, United States of America; 3 Department of Biology, Colorado State University, Fort Collins, Colorado, United States of America; KIT, GERMANY

## Abstract

The data collected by mobile methane (CH_4_) sensors can be used to find natural gas (NG) leaks in urban distribution systems. Extracting actionable insights from the large volumes of data collected by these sensors requires several data processing steps. While these survey platforms are commercially available, the associated data processing software largely constitute a black box due to their proprietary nature. In this paper we describe a step-by-step algorithm for developing leak indications using data from mobile CH_4_ surveys, providing an under-the-hood look at the choices and challenges associated with data analysis. We also describe how our algorithm has evolved over time, and the data-driven insights that have prompted these changes. Applying our algorithm to data collected in 15 cities produced more than 6100 leak indications and estimates of the leaks’ size. We use these results to characterize the distribution of leak sizes in local NG distribution systems. Mobile surveys are already an effective and necessary tool for managing NG distribution systems, but improvements in the technology and software will continue to increase its value.

## Introduction

Mobile atmospheric methane (CH_4_) analyzers have been developed as an effective tool for identifying natural gas (NG) leaks in urban distribution systems [1,2]. The advantages of these highly-sensitive instruments on a mobile platform include the ability to detect more leaks and to quickly survey large spatial regions. These benefits are prompting many local distribution companies to adopt this technology as an integral part of managing their NG distribution system. For example, information from these surveys has been used by local distribution companies to prioritize millions of dollars in pipeline replacement [3,4]

A crucial and challenging step in using this monitoring technology is translating raw survey data into actionable information about natural gas leaks. The instruments used in these surveys produce large volumes of data, including atmospheric CH_4_ concentrations and GPS locations, as well as wind speed and direction, current time, instrument functionality, and vehicle speed, all at 2 Hz. From this stream, an algorithm is needed to generate actionable information such as locations of natural gas leak indications and an estimate of each leak’s emission rate.

Algorithms for processing data from mobile CH_4_ surveys have been commercially developed [5,6], but these algorithms are proprietary information, and little is known about the details of the processing steps. In this paper, we describe the algorithm and processing steps that we use to develop leak indications from mobile CH_4_ surveys. Collectively, we refer to our data processing steps as our leak indication algorithm. Hereafter, unless specified otherwise, we use the term algorithm to mean leak indication algorithm.

An algorithm for processing mobile CH_4_ survey data requires multiple steps. Within each step, the user will need to make data processing decisions. For example, when are CH_4_ concentrations large enough to flag as a leak indication? While these decisions are subject to trade-offs (e.g., sensitivity and specificity), we use data, validation, and in-field experience to inform our processing protocols whenever possible.

The goal of our algorithm is to convert raw survey data about atmospheric CH_4_ concentrations into data products that contain actionable information to be shared with local distribution companies (LDCs) and the public. The data products derived from these surveys can be customized to fit the user’s need, and there is great potential for the development of new data products. Here we describe how we develop a map of natural gas leak indication locations and estimates of the leaks’ emission rate. We also describe a method for identifying especially leaky areas of NG pipe, illustrating the potential for deriving multiple data products from these surveys.

Since its initial implementation as detailed in von Fischer et al. [1] referenced here as v1.0, our algorithm has undergone several changes to improve its efficacy, leading to v2.0 presented here. Here we provide more details about the algorithm and describe updates to the algorithm while presenting the data that motivated and supported these updates. Finally, we present a summary of the database of leak indications that we’ve derived from surveying and applying our algorithm in multiple cities. The rest of the paper is organized as follows: in Section 2 we describe our algorithm and updates we have made to it; Section 3 compares the results from applying v. 1.0 and 2.0 of our algorithm to survey data from four cities; in Section 4 we provide an analysis of our leak indication database derived from surveying in multiple cities; Section 5 concludes the paper with a discussion.

## Survey procedures and leak indication algorithm

**Fig 1:**
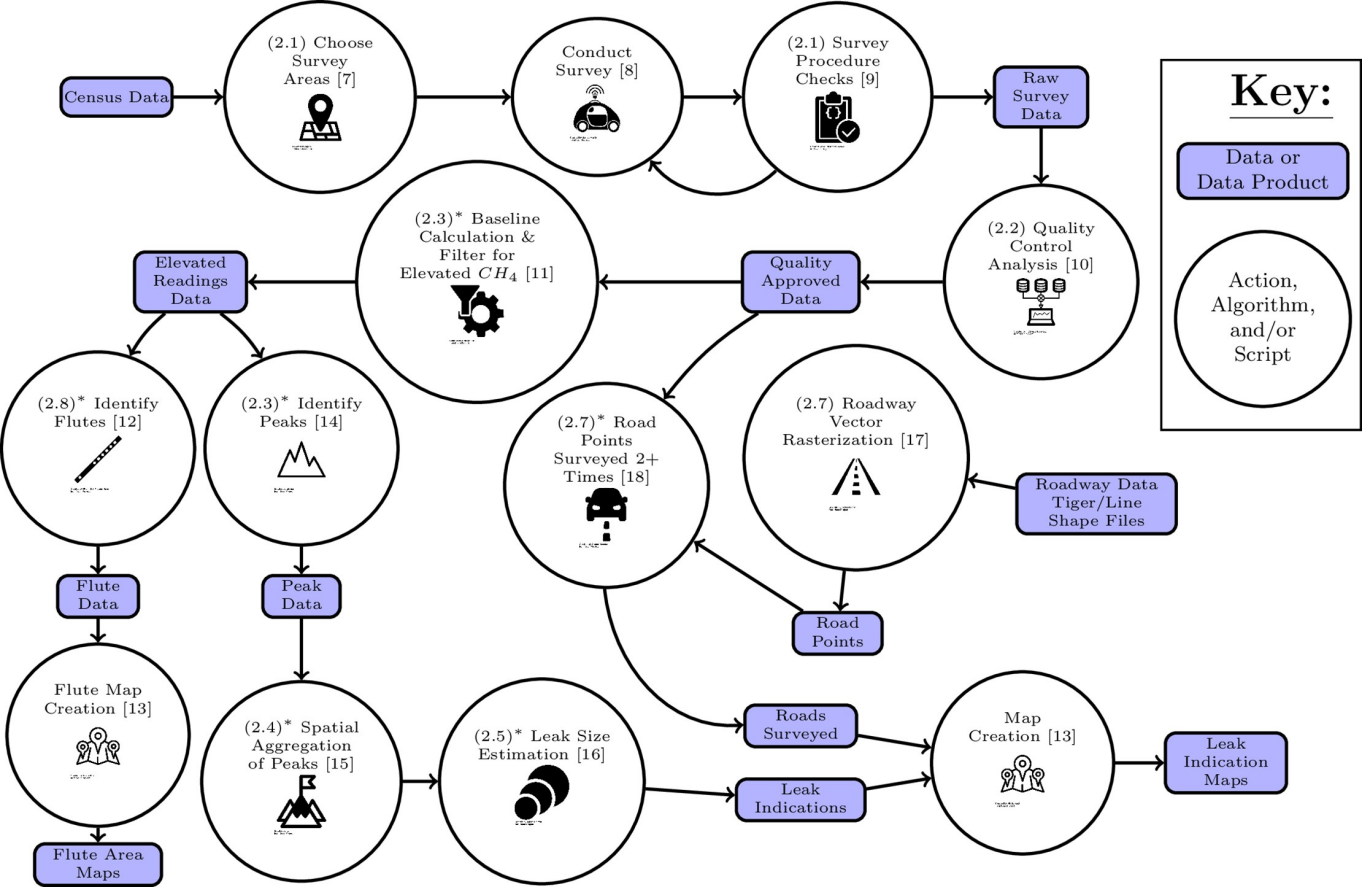
Flow chart of the steps used to create maps of leak indications and survey coverage. The parenthetical numbers denote the section of the paper that describes the data processing step. Topics marked with an asterisk (*) denote parts of the algorithm that have been modified since v1.0. Icons are reprinted from The Noun Project under a CC BY license, with permission from icon authors. Individual author attributions are given in the references **[7–19]**.

In this section we describe the survey procedure for the mobile CH_4_ surveys, and the steps of the algorithm that we use to extract actionable information from the survey data. As we illustrate in Fig 1 there are a number of stages in the survey procedures and the steps of data processing. We organize the text below to follow the stages outlined in the figure above. Table 1 lists and describes the data used in our algorithm and the data products it produces. We refer the interested reader to von Fischer et al. [1] for a description of the sampling instrumentation, and further details about the controlled CH_4_ releases and decisions made in the data processing algorithm.

**Table 1:** Summary of the data used by our algorithm and the data products it produces.

Data/Data Product	Data type	Section	Description, Attributes, and/or Key Features
Census Data	Input	2.1	American Community Survey demographic information
Tiger/Line Files	Input	2.7	Roadway location information, vector form
Raw Survey Data	Input	2.1	Vehicle location, time stamp, atmospheric CH_4_ concentration, quality control metrics
Road Points	Derived Intermediate	2.7	Roadway location information, raster form
Quality Approved Data	Derived intermediate	2.2	Survey data filtered for QC standards and adjusted for GPS-sampling delay
Elevated Readings Data	Derived intermediate	2.3	Baseline CH_4_ concentration; locations, time stamp, and CH_4_ concentration for readings over baseline
Observed Peak Data	Derived intermediate	2.3	Central location, time stamp, and CH_4_ concentration for each set of elevated readings
Leak Indications	Derived final	2.4	Aggregations of peaks: estimated location of NG leak, max CH_4_ observed, first and last date peaks were observed, estimate of emission rate
Roads Surveyed	Derived final	2.7	Roadway locations that have been surveyed two or more times
Leak Indication Maps	Combination of derived final	—	Visualization combining roads surveyed and leak indications
Anomalous Leak Indications	Derived final	4.2	Locations of unusually large peaks or emission rates
Flute data	Derived final	2.8	Aggregation of multiple observed peaks in close proximity: estimated location of extended area with high density of leak indications.

### 2.1 Survey procedure

In most cases, whole cities were not mapped exhaustively. Instead, we identified subsets of the city to serve as survey regions. In some cases, our interactions with local distribution companies directed our survey toward specific regions of interest. In other cities, survey regions were chosen to reflect the variation in socio-economic conditions, housing age (intended as a proxy for NG pipeline age) and demographic composition of the city, based on data obtained from the U.S. Census Bureau [20] and Google Maps.

Our survey protocols have not changed since v1.0, and here we further describe how these protocols were implemented. We instructed the drivers of the survey vehicle to drive every roadway within each designated survey region. The driver was able to monitor their coverage of the region via a display screen in the car. Data from each day were uploaded to a data cloud. As coverage of each region neared completion, we checked for survey gaps. We defined gap areas as any roadway segment(s) in the survey region where either the driver failed to survey or where data quality was poor (see Section 2.2 for more information on data quality). If we found gap areas, we sent a map of these areas to the driver, instructing them to drive the survey vehicle through these areas. Due to idiosyncratic issues (e.g., private roads, road construction, driver error, data quality) that arose during surveying, spatial coverage of survey regions was not always 100%, but was typically >90%. Once a survey region had been surveyed once, we designated the region as having the first pass completed. We then instructed the car driver to survey the region a second time (second pass), using the same protocols as the first pass. Completion of each pass of a survey region typically took 2–3 days but occasionally did take longer due to size of the region, mechanical failures, weather, and/or traffic.

During data collection, we ran checks for quality control, instrument error, and survey progress every 1–2 days. We ran checks for anomalous leaks (see Section 4.2) every 1–2 weeks. Collecting data for all the survey regions within a city typically took several months. Final processing of all the data collected in a city took place after data collection in all survey regions was complete.

### 2.2 Quality control and GPS adjustment

We use a number of quality control (QC) checks to ensure that the survey data are reliable and to ensure the instruments are functioning correctly. As described in v1.0, we do not use data collected by the car when the car speed is greater than 20 m/s (45 mph); controlled CH_4_ releases [1] indicated that CH_4_ concentration measurements become unreliable at high vehicle speeds. Instrument performance metrics, including inlet pressure, cavity temperature, and instrument temperature were defined by the instrument manufacturer. Deviations from these normal operating ranges are possible indications of a problem with the sampling system.

In reviewing QC data from two cities, we found that 9% and 7% of data collected were discarded due to failing to meet QC criteria. The most commonly failed QC criteria was vehicle speed, which accounted for the vast majority of discarded data. Although survey areas generally did not include high-speed roadways, the drivers often commuted from their home to the survey region via highways, and the instruments collect data whenever the car is running. Low cavity pressure, an indicator of a clogged sampling inlet, was the next most commonly failed QC criteria.

Because there is a delay in the time between when air is sampled at the front bumper and when it is analyzed by the instrument, we adjust GPS locations to account for this delay. This adjustment is described in algorithm v1.0. As an example, if the GPS-sampling time delay is 2 seconds, latitude and longitude coordinates are reassigned to CH_4_ readings that were observed two seconds previous.

### 2.3 Peak detection

The first step in identifying NG leak indications is to characterize departures from typical levels of CH_4_. We characterize these departures by first defining a baseline (or background) CH_4_ concentration. Atmospheric CH_4_ concentrations are typically around 2 parts-per-million (ppm), but they vary at the small, localized scale where CH_4_ measurements are taken during a mobile survey. Thus, it is necessary to define a baseline concentration as a function of local CH_4_ measurements. We calculated a baseline concentration for each CH_4_ measurement made by the survey instrument.

We updated our baseline calculation methodology between v1.0 and v2.0 of our leak detection algorithm. We now define the baseline concentration associated with a CH_4_ measurement as the median of all CH_4_ concentrations recorded within a 2.5 minute window of the reading (i.e., the median of all readings that occurred 2.5 minutes before and 2.5 minutes after the given reading). Previously, we defined the baseline for each CH_4_ measurement as the average of all CH_4_ readings that occurred in the previous 2 minutes of surveying. An analysis of our baseline CH_4_ concentration data revealed that, when the survey vehicle drove through the gas plumes from multiple leaks that were spatially close, these baseline concentrations tended to become inflated (e.g., larger than 3.5 ppm). This inflation hinders our ability to detect the departures from typical CH_4_ levels caused by NG leaks. The +/- 2.5 minute window allows a greater spatial extent of methane concentrations to be used for characterizing the baseline relative to using only the data from the previous 2 minutes. The median provides a better measure of typical CH_4_ concentrations than the mean because it is relatively unaffected by the large CH_4_ concentrations that occur when driving through the plumes created by NG leaks.

**Fig 2:**
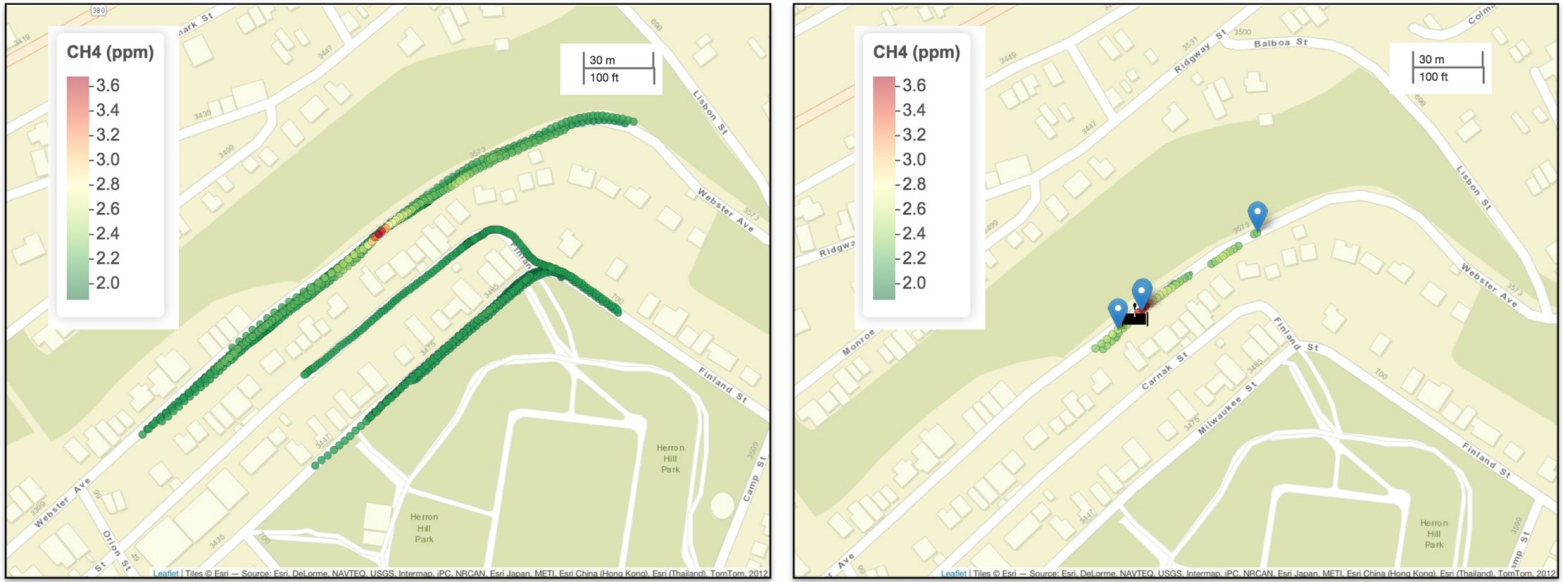
An example of data processing from the CH_4_ survey. The left panel shows the survey data after quality control. The right panel displays the elevated readings, observed peaks (blue markers), and leak indication (black pipe marker) information within the black rectangle marked in the left panel. The leak icon is reprinted from The Noun Project under a CC BY license, with permission from icon authors. Individual author attribution **[19]** is given in the bibliography. Maps were created using Leaflet for R **[21]**.

Next, we define a threshold for elevated CH_4_ concentrations using the baseline value. Unchanged from v1.0 of the algorithm, we define an elevated reading as any reading having CH_4_ levels greater than or equal to 110% of the baseline value. Because the baseline value will vary in time and space, so will the threshold for elevated CH_4_ levels, but at a typical background of 2 ppm, the threshold is 2.2 ppm. Fig 2 displays an example of survey data where we found elevated CH_4_ readings.

The percentage of readings that are considered elevated varied by city and reflect the relative leakiness of NG infrastructure. For example, in one city known to have relatively large amounts of leak prone pipe, 1.8% of CH_4_ readings were marked as elevated. In another city, which has relatively newer infrastructure, 0.4% of reading were marked as elevated.

When the survey vehicle drives though a plume of gas created by a NG leak, it is typical to observe multiple elevated readings in series. Plots of these elevated CH_4_ measurements as a function of time reveal a peak shape, where concentrations rise and fall as the car passes through the plume. We refer to these consecutive elevated readings as observed peaks (OPs). Each OP consists of a central location, maximum CH_4_ concentration, and timestamp of observation. The central location of an OP is defined as a weighted spatial average of the locations of all the elevated readings in the OP, where the weights are defined using the corresponding CH_4_ measurements. The OP location is given by

$$\left(\mathrm{Long},\mathrm{Lat}\right)=\frac{\sum_{i=1}^{n}w_{i}\left({\mathrm{long}}_{i},{\mathrm{lat}}_{i}\right)}{\sum_{i=1}^{n}w_{i}},$$ (1)

where w_i_ is the CH4 concentration recorded from the i^th^ elevated reading and n is the number of elevated CH_4_ readings in the peak.

OPs are an indicator that a natural gas leak or other CH_4_-emitting source is present near-by. In some cases, CH_4_ levels can change from elevated to non-elevated to elevated within a short time period. If the time between two series of elevated readings is less than 5 seconds (i.e., if readings go from elevated to non-elevated to elevated within 5 seconds), the two series are treated as a single OP. In v1.0 when two such series were observed, they were treated as two separate OPs regardless of the time between observations. In some cases, these two OPs would be joined into a single leak indication (see Section 2.4) despite elevated methane levels being observed on only a single day. The 5 second interval is based on a driving speed of 11–13 m/s (25–35 mph), so that 5 seconds of driving equates to a distance of approximately 55–67 meters.

### 2.4 Leak indications

The next step of the algorithm is consolidating OPs into leak indications. When multiple OPs are located in close spatial proximity, they likely correspond to detections of the same CH_4_-emitting source. Environmental variables, small-scale winds or soil conditions, can change the apparent location of a CH_4_-emitting source. To account for this change in apparent location, we place a spatial buffer around each OP location and join OPs with overlapping buffers. In algorithm v1.0, we used a 20 m buffer for the join. Data from our field work with one utility company revealed multiple instances where observed peaks from the same leak were separated by more than 20 meters. These data suggested that a 30 m buffer was more appropriate, and as a result, we use a 30 m buffer to join OPs in algorithm v2.0.

We join OPs that have overlapping buffers into a single leak indication location. Thus, a leak indication corresponds to an area where elevated CH_4_ levels have been observed two or more times. Each leak indication consists of a location (lat/long), first and last date of observation, the number of OPs joined to create the leak indication, and an estimated emission rate (see Section 2.5 for more information on estimated emission rates).

The percentage of OPs that become a part of a leak indication varied by city and again reflected the state of infrastructure in the city. In the aforementioned city known to have a relatively large proportion of leak prone pipe, 75% of OPs became part of a leak indication. In the other city, which has relatively newer infrastructure, 47% of OPs became part of a leak indication. These percentages also suggest that leaks are larger in the first city than the second, as the probability of detecting a leak increases with leak size [22]. Leak indications typically consist of 2 or 3 OPs (detections), although it is not unusual to consolidate as many as 5–8 OPs into a single leak indication. Occasionally, when located along arterial routes crucial for navigation or near the driver’s residence, leaks may be detected as many as 15+ times.

Following algorithm v1.0, we do not report locations where elevated CH_4_ levels (i.e., an OP) have only been detected once. We require repeated observation of elevated CH_4_ levels in order to reduce false positive reporting where no leak is present. Transient sources of CH_4_ emissions such as compressed natural gas vehicles can cause elevated CH_4_ levels to be detected by our mobile surveyor. The tradeoff for reducing false positives is an increase in false negatives. Leaks can go undetected for a variety of reasons including wind (e.g., wind blowing the plume away from the roadway) and soil conditions (e.g., wet soil preventing the escape of gas). As a result, the leak indications reported from our survey efforts are a subset and not a census of all leaks present in the distribution system. See also Weller et al. [22] for a discussion of this phenomenon.

New to algorithm v2.0, we calculate the location of leak indications using a weighted spatial average of the locations of the OPs that compose the leak indication. The location of a leak indication is an estimate of the location of a potential natural gas leak. In algorithm v1.0 we used an unweighted spatial average. Each OP has a maximum recorded CH_4_ concentration and a location associated with this maximum concentration. When OPs are joined, we average the coordinates of the maximum CH_4_ concentration from each peak. Coordinates with the largest CH_4_ concentrations are given the most weight. We use a weighted average because data from controlled CH_4_ releases showed that, for a given leak size, the largest measured CH_4_ concentrations are more likely to be observed when the vehicle is closest to the expression point. The final estimate of the leak’s location is given by:

$$\left(\mathrm{Long},\mathrm{Lat}\right)=\frac{\sum_{j=1}^{p}w_{j}\left({\mathrm{long}}_{j},{\mathrm{lat}}_{j}\right)}{\sum_{j=1}^{p}w_{j}},$$ (2)

where w_j_ is the maximum CH_4_ concentration from the j^th^ observed peak and p is the number of OPs that were joined to create the verified peak.

**Fig 3:**
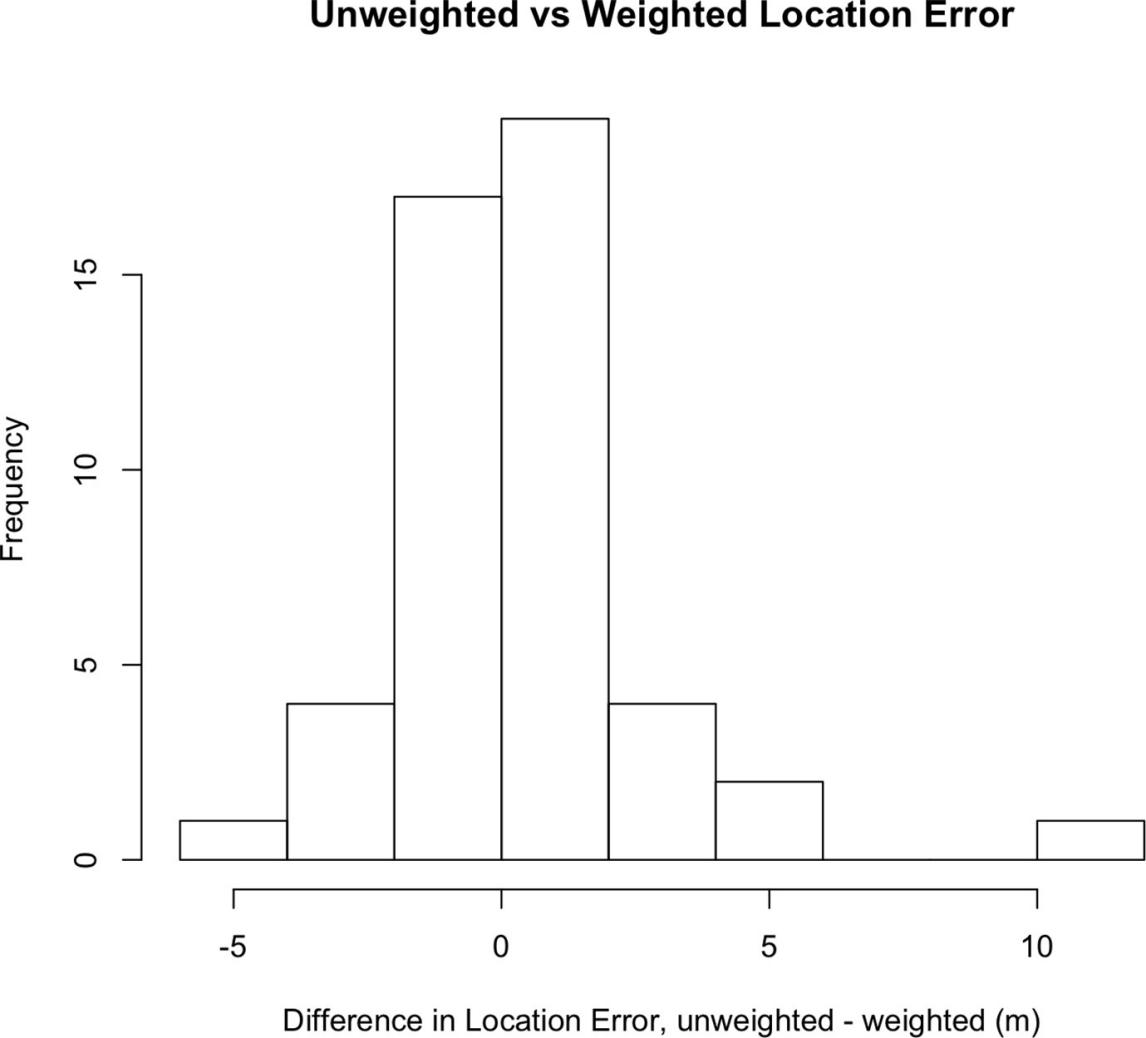
Histogram of the difference in location error. The difference in location error is defined as unweighted error–weighted error. Positive values of this difference indicate that the weighted location estimate was closer to the actual leak. The mean difference was 0.38 m, and the weighted location was closer for 54% of the sites (26/48).

We used a handheld GPS to record the coordinates of leaks during field work in one city to assess whether or not our weighted spatial average produced improved leak location estimates. We compared the leak location error associated with the weighted and unweighted location estimates. The location error is defined as the distance between the estimated leak location and the actual leak location recorded in the field. The difference in the location error is defined as the unweighted location error minus the weighted location error. Thus, positive values of this difference indicate that the weighted location estimate was closer to the leak. A histogram of the differences is show in Fig 3. The average of this difference was 0.38 m, and the weighted location was closer to the actual leak for 54% of the leaks (26/48). In one instance it was 11 m closer, although the absolute difference tends to be small (less than 5 m). In another study [2] we found that the weighted location formula reduced the location error by an average of 1.8 m. Thus, while this change tends to produce better location estimates, the improvement is modest and could be included as a user-specific option.

### 2.5 Estimating emission rates

We updated the way we estimate leak emission rates relative to algorithm v1.0. In an examination of our leak rate prediction approach, we identified a tendency to over-estimate the leak rate, some statistical collinearity among predictors, and inconsistent choice of data to use in the calibration. As a result, we revisited the controlled CH_4_ release data and analysis described in v1.0. The resulting changes include: 1) removal of some calibration data, 2) change in the elevated CH_4_ features used to predict leak rate, and 3) an updated maximum estimated leak rate.

We use a statistical calibration model for estimating leak size using data from controlled CH_4_ releases. At low release rates and large distances, we sometimes failed to detect departures from the baseline CH_4_ concentration (i.e., we did not observe concentrations that were greater than 110% of the baseline). In this new analysis, we removed passes from the calibration experiment where we failed to detect elevated CH_4_ levels; the previous calibration included some passes where the CH_4_ readings were elevated but did not rise above 110% of baseline.

**Fig 4:**
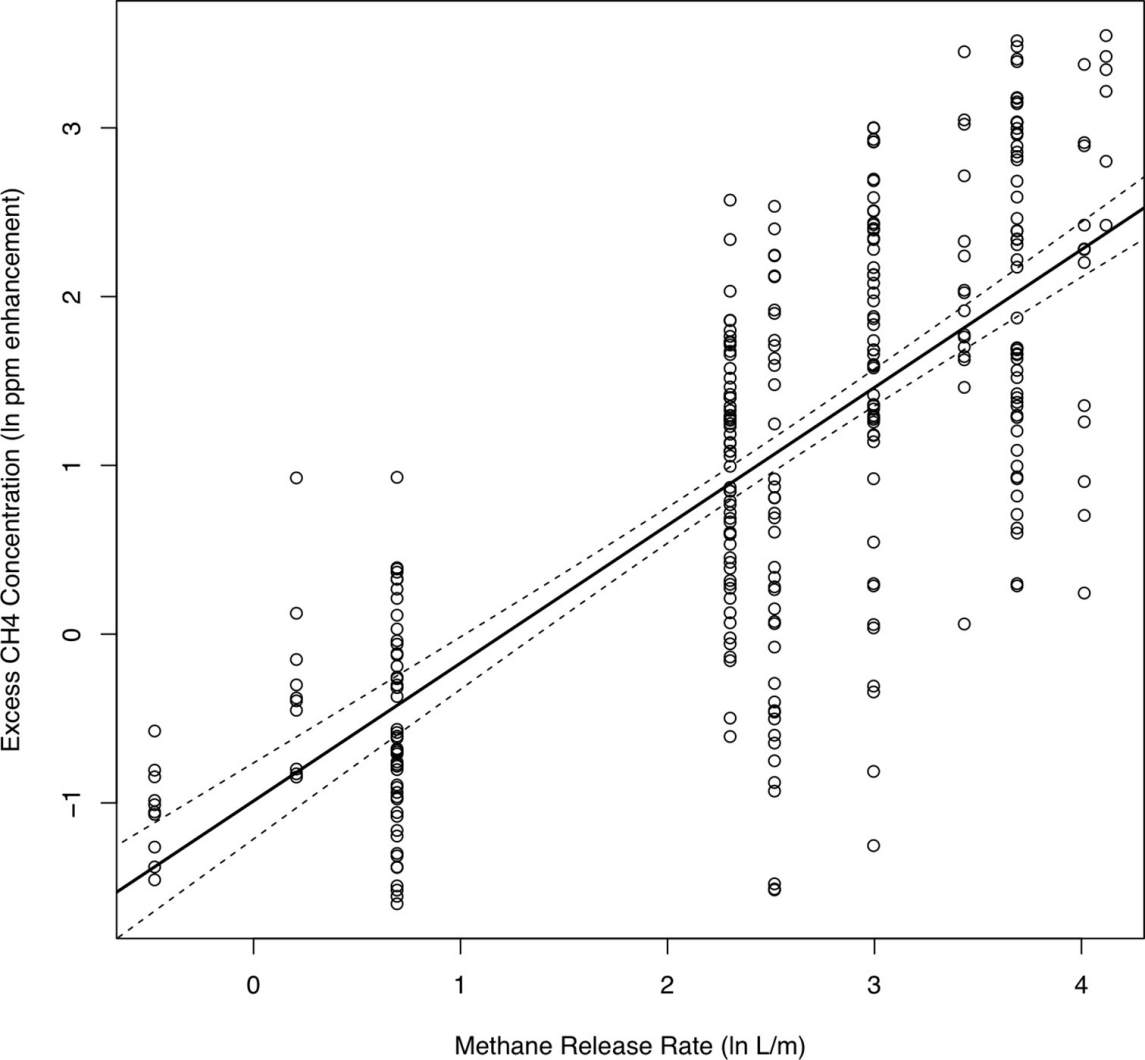
Relationship between known emission rate and excess CH_4_ from controlled release experiments. The axes are on the ln-ln scale.

An exploration of the controlled release data using R software [23] indicated that the maximum CH_4_ enhancement from each pass of the controlled release was the best predictor of the leak emission rate, and that additional predictors did not meaningfully improve model predictions. We define the CH_4_ enhancement, or excess CH_4_, as the difference between the measured CH_4_ concentrations and the baseline concentration. The relationship between the known emission rate and the maximum excess CH_4_ from the controlled release experiments is illustrated in Fig 4. As expected, the observed maximum excess CH_4_ tends to increase as the CH_4_ release rate increases.

**Fig 5:**
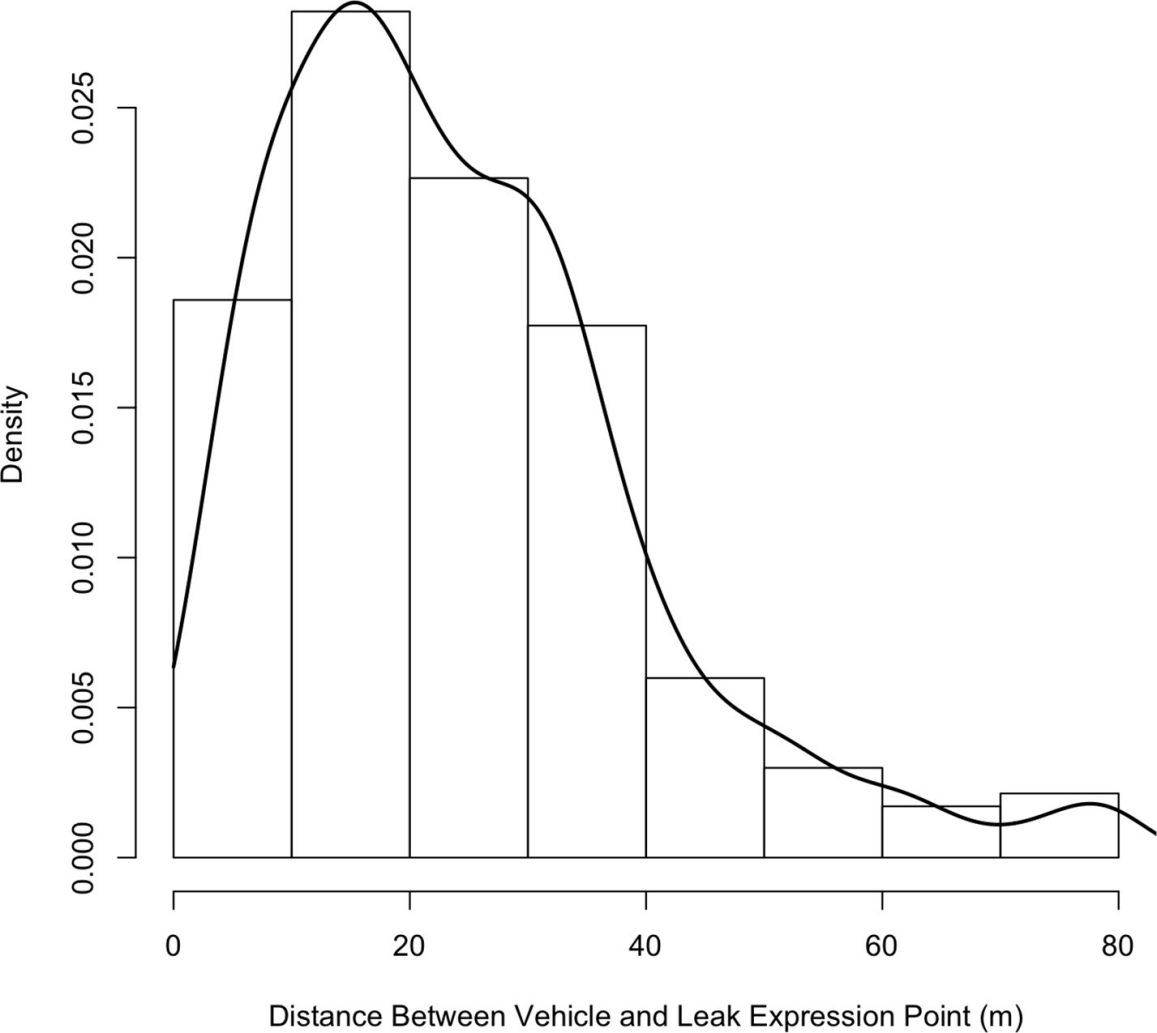
Histogram of the distance between the survey vehicle and NG leak expression points in Boston. In this histogram, there were 468 passes of 19 known leaks. The typical distance between the car and leaks is 10–30 meters. Occasionally, the survey instruments detect leaks that are greater than 60 m from the car.

Besides the leak emission rate, our analysis of the controlled release data indicated that distance between the car and the leak expression point is also an important factor in understanding the relationship between the release rate and the excess CH_4_ concentrations. In practice, however, we do not know the distance between the car and the leak expression point. Thus, we do not include distance in our subsequent analysis. Instead, our calibration analysis assumes an average distance of 15.75 meters between the car and the leak. Data on the distance between the survey vehicle and known leak locations indicated that the typical distance between the car and detected leaks was 21 meters (see Fig 5).

We fit a linear regression model to the controlled release data, using natural log (*ln*) of the known release rate as the explanatory variable (x) and the *ln* of the maximum excess CH_4_ as the response variable (y). We use the model

$$ln(\max excess\;CH_{4})=b_{0}+b_{1}*ln\left(emission\;rate\right)+\varepsilon ,\varepsilon {∼}^{iid}N\left(0,\sigma^{2}\right),$$ (3)

where the emission rate is given in L/min and the excess CH_4_ is given in ppm. The estimated regression equation is

$$\ln(\max excess\;CH_{4})=-0.988+0.817*\ln\left(emission\;rate\right).$$ (4)

To use this model to estimate the emission rate of a leak in practice, we calculate the geometric mean of max excess CH_4_ values associated with each detection of the leak. Recall, each leak indication results from two or more observed peaks, each of which has a *ln*(max excess CH_4_) value. We enter the average *ln*(max excess CH_4_) into the left-hand side of Eq (4) and solve for the emission rate.

### 2.7 Roads driven two or more times

Among the data products that we generate, we provide maps showing locations where the survey vehicle has collected useable survey data two or more times. This map is derived using U.S. Census TIGER/Line GIS (Geographic Information System) [24] files to define the locations of roadways within the survey regions. We rasterize the relevant roadway vectors, discretizing them into road points placed 20 m apart along the roadway vector.

We then use ArcGIS to analyze the GPS coordinates and time stamps of processed survey data in order to determine how many times a road point has been surveyed. We define a road point as having been surveyed if the survey vehicle passed within 20 m of the road point. This 20 m buffer accounts for GPS and/or GIS road location errors. In algorithm v2.0 we define a road point as driven two or more times if two or more survey attempts occurred at least 30 seconds apart in time. Previously we required survey attempts to be separated by five minutes.

Due to the logistics of driving in an urban area, many roadways are surveyed more than two times. We similarly define a road point as having been driven three times, four times, etc. In the overwhelming majority of cases, road points driven 2+ times have been surveyed on at least two separate days. We estimate the number of miles driven 2+ times by interpolating adjacent road points that were driven 2+ times using ArcGIS. Table 2 displays the number of road miles driven and number of survey attempts from two cities.

**Table 2:** The number of road miles driven by survey effort in two cities.

	2	3	4	5	6	7	8	9+
Jacksonville	80	137	156	126	85	61	47	48
Pittsburgh	300	183	139	112	70	72	62	35

### 2.8 Flutes

In some cities, leak prone pipeline infrastructure (e.g., cast iron pipe) can develop multiple leaks in close proximity. We refer to these leaky segments of pipe as flutes because, like the musical instrument, they have multiple gas escape routes along their flow path. These leaky segments of pipe can cause elevated CH_4_ levels to be detected over a large spatial extent. Our leak indication algorithm typically cannot resolve these individual leaks due to their close proximity; instead, they are often consolidated into a single leak indication.

**Fig 6:**
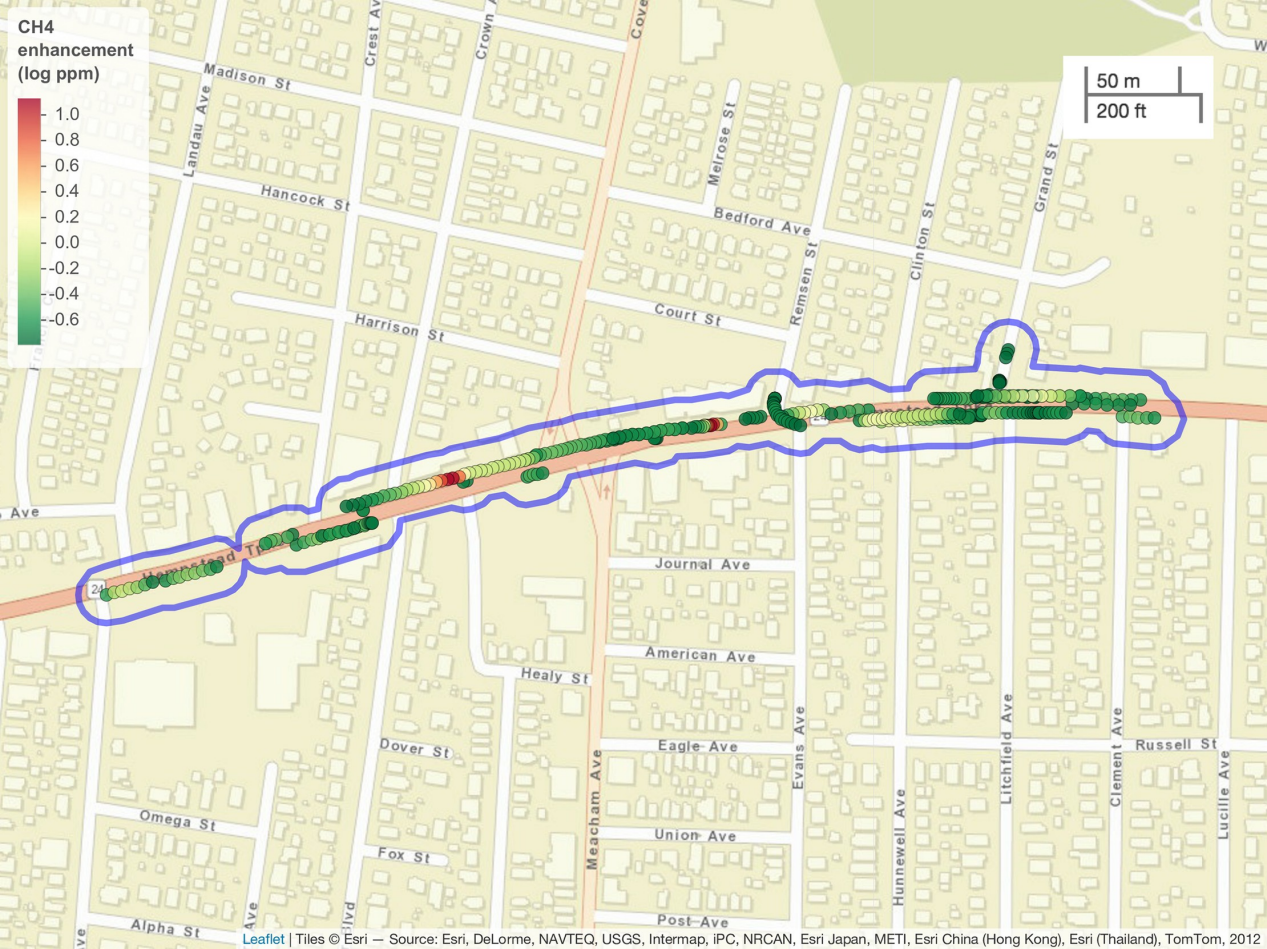
An example of a flute area (blue line). Segments of pipe with multiple leaks can cause elevated CH_4_ levels to be detected over a large spatial extent, as illustrated by the blue polygon encircling multiple series of elevated readings. The scale is the log of the CH_4_ enhancement (log_10_ ppm over baseline). Map created using Leaflet for R **[21]**.

Flute areas highlight locations that contain an unusually high frequency of elevated CH_4_ concentrations over a longer stretch of roadway than a leak indication. We identify these areas by buffering and then joining together the locations of elevated CH_4_ readings. By joining these buffered locations, we estimate the area of the spatial extent of elevated CH_4_ levels. We can then use these size estimates to rank and identify locations that may contain multiple leaks. Fig 6 shows an example.

Identification and ranking of flute areas can be used to prioritize more intensive effort of pipe segment replacement rather than individual leak repair. When we shared our flute areas with one LDC, they indicated that the maps would be useful, and they noted that two of the five flute areas that we identified had already been slated for >1 mile of pipeline replacement. We do not yet have a mechanism for estimating total gas emission rates from flutes.

## Analysis of algorithm modifications

The most significant updates leading to algorithm v2.0 include the baseline calculation and the method used to estimate leak emission rate. Smaller changes include the threshold for joining observed peaks into leak indications, timing between observed peaks, and timing used to calculate roads driven two or more times. Although no single change to our algorithm is dramatic, we anticipated that the combination of these changes could have significant effect on the conclusions we draw from the data. Here we compare findings of the two algorithms in light of this potential. The results of this analysis are displayed in Table 3. One of the cities used in the analysis is anonymized because the results have not yet been shared publicly.

**Table 3:** A comparison of survey results between algorithm v1.0 and v2.0.

City	Counts & Miles	v2.0	v1.0	Emissions	v2.0	v1.0
Birmingham	Observed peaks	728	654	Sm	159	141
	Leak indications	168	160	Med	7	19
	Miles 2+	366	354	Lg	2	0
	LI/Mile	0.46	0.45	Total emission (L/min)	592	648
				L/min/Mile	1.62	1.83
Anonymous City	Observed peaks	1115	1075	Sm	251	215
	Leak indications	275	277	Med	24	59
	Miles 2+	569	488	Lg	0	3
	LI/Mile	0.48	0.57	Total emission (L/min)	774	1636
				L/min/Mile	1.36	3.35
Pittsburgh	Observed peaks	2306	2041	Sm	422	374
	Leak indications	460	447	Med	35	69
	Miles 2+	1532	1347	Lg	3	4
	LI/Mile	0.30	0.33	Total emission (L/min)	1419	2781
				L/min/Mile	0.93	2.06
Dallas	Observed peaks	2066	1856	Sm	390	353
	Leak indications	414	414	Med	23	57
	Miles 2+	875	873	Lg	1	4
	LI/Mile	0.47	0.47	Total emission (L/min)	1226	1913
				L/min/Mile	1.40	2.19

As expected, v2.0 of the algorithm produces more OPs because the baseline concentration tends to be less affected by areas with elevated CH_4_ concentrations. This allows us to more easily detect small departures from baseline. Surprisingly, the increase in observed peaks does not always lead to an increase in leak indications. This is in part due to the larger buffer used around OPs in v2.0, which consolidates more OPs together. For example, the larger buffer could join five OPs into a single leak indication rather than two separate leak indications. In two of the cities the number of leak indications increased while in the other two the count stayed the same or slightly decreased.

Despite changes in the number of leak indications and miles of roadway surveyed, the density of leak indications per mile was relatively stable across cities between v1.0 and v2.0 (Table 3). Across the 15 cities we have mapped thus far, we have observed a range in leak indication density from 0.005 to 1 leak indications per mile). In light of this ~200-fold range, the <20% change in leak indication density arising from algorithm changes (Table 3) does not meaningfully alter the qualitative comparison among cities. Thus, we conclude that the leak indications per mile metric can be fairly compared between cities, regardless of whether v1.0 or v2.0 was used to analyze the cities’ data.

In a related study, we evaluated the precision and accuracy of our updated model for estimating emission rates by comparing it with enclosure and tracer release quantification methods [2]. This comparison revealed that although the mobile quantification method still over-estimates the size of the smallest leaks, it is a generally unbiased estimator of leak size for the larger leaks that are of primary interest for pipeline management. We explored the impact of the updated model on estimated emission rates.

The updated emission rate model generally predicted smaller rates. As a result, the estimated total emissions decreased in all four cities, but, once again, the magnitude of the decrease varied by city. For Anonymous, Pittsburgh, and Dallas, the v2.0 estimate of total emissions was about 46% less than the v1.0 estimate. In Birmingham, v2.0 estimated emissions were only 9% lower than v1.0. Estimates of emission rates per mile of roadway similarly decreased. In Anonymous City and Pittsburgh, estimates of liters per minute per mile were reduced by roughly 57%. In Dallas the reduction was 36%, and in Birmingham it was 11%. For the cities considered here, the liters per minute per mile only varied by an order of 1.7x, from 0.93 to 1.62.

To account for uncertainty in leak size estimation, we assign leaks to size bins (small, medium, and large), following the same categorization as v1.0. The reduction in estimated emission rates from v1.0 to v2.0 caused an increase the number of leak indications that were categorized as small, and typically, a decrease in the number of leak indications categorized as medium or large. An exception to this was Birmingham, where the number of large leaks increased from zero to two, largely due to the updated baseline calculation and a number of large OPs in close proximity that had previously been joined into fewer large leak indications.

## Survey efforts and anomalous leaks

### 4.1 Leak indication database

Cumulatively, our survey efforts between 2013 and 2017 have covered over 13,300 miles of roadway across 15 cities. This survey provides information about natural gas leak indications in 1405 census tracts, home to an estimated 5.7 million people [25]. By applying our algorithm to the data collected in these 15 cities, we have developed location and emission estimates for 6125 leak indications. This database will grow as we complete our survey efforts in 2018. Leak indication maps and data are publicly available on the EDF website.

**Fig 7:**
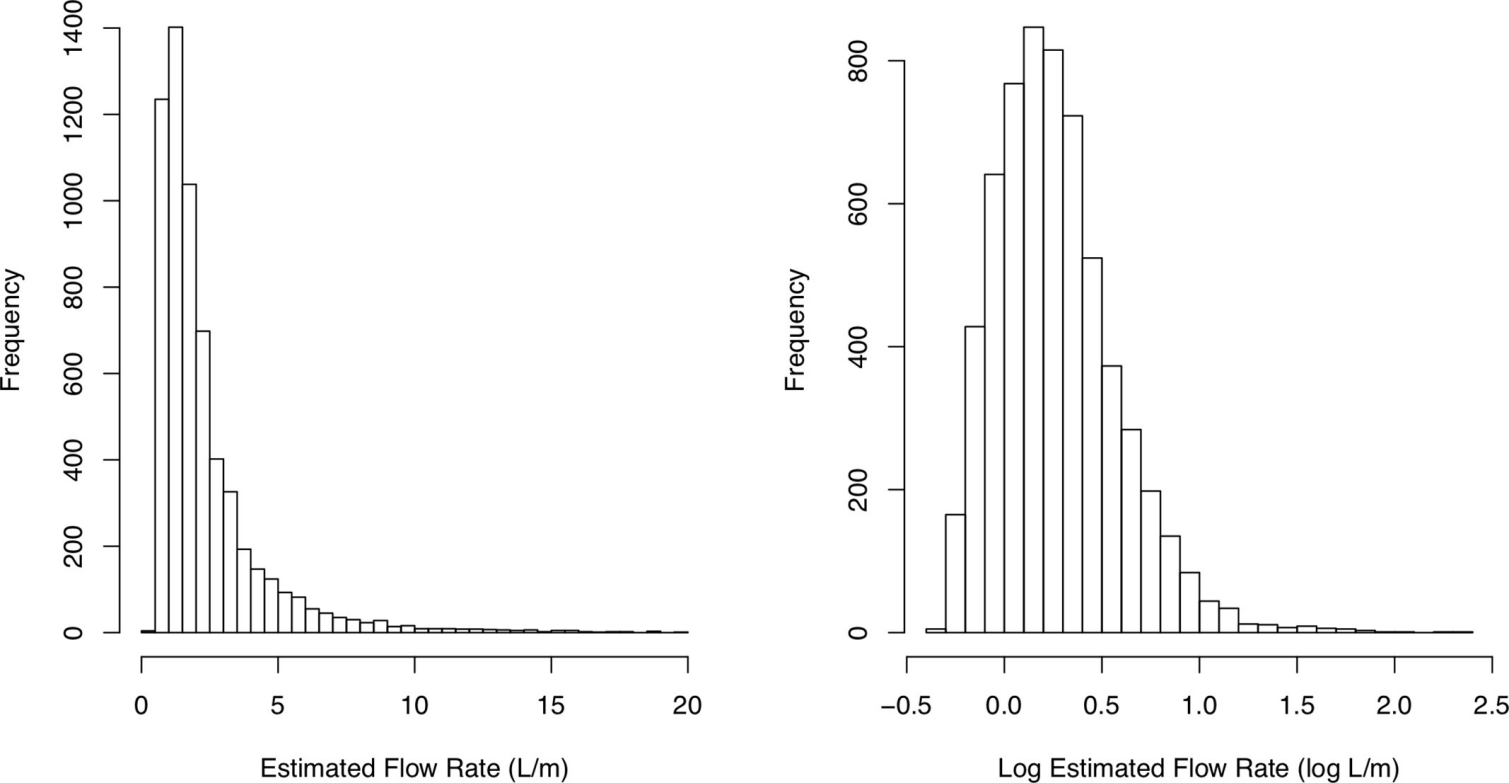
Histograms of estimated sizes of 6125 leak indications developed from surveying in 15 cities. The left figure shows a histogram of estimated emission rates truncated at 20 L/min. The right figure shows a histogram emission rates from all 6125 leak indications on the log_10_ scale.

Fig 7 shows a histogram of estimated emission rates for the 6125 leak indications. The shape of the distribution of estimated leaks sizes is typical of other studies that quantify CH_4_ emissions from the NG production and distribution process [26–28]. We see a right-skewed distribution where most of the leaks are small but there are a number of leaks with exceptionally large emission rates. The smallest estimated emission rate was 0.47 L/min and the largest was 238 L/min. This highest value is an extrapolated estimate beyond the range of our calibration experiment, which used a maximum release rate of 61.5 L/min. Of the 6125 leak indications, only 7 were estimated to be greater than 61.5 L/min. For the bin sizes, 93.8% of the leak indications were categorized as small, 5.9% were medium, and 0.3% were large. The estimated total emissions from these leak indications are 16,261 L/min. Assuming a 20-year global warming potential of 84 for CH_4_ [29], the daily emissions from these leaks are equivalent to 1286 metric tons of CO_2_, roughly the CO_2_ emissions from burning 703 tons of coal [30].

**Fig 8:**
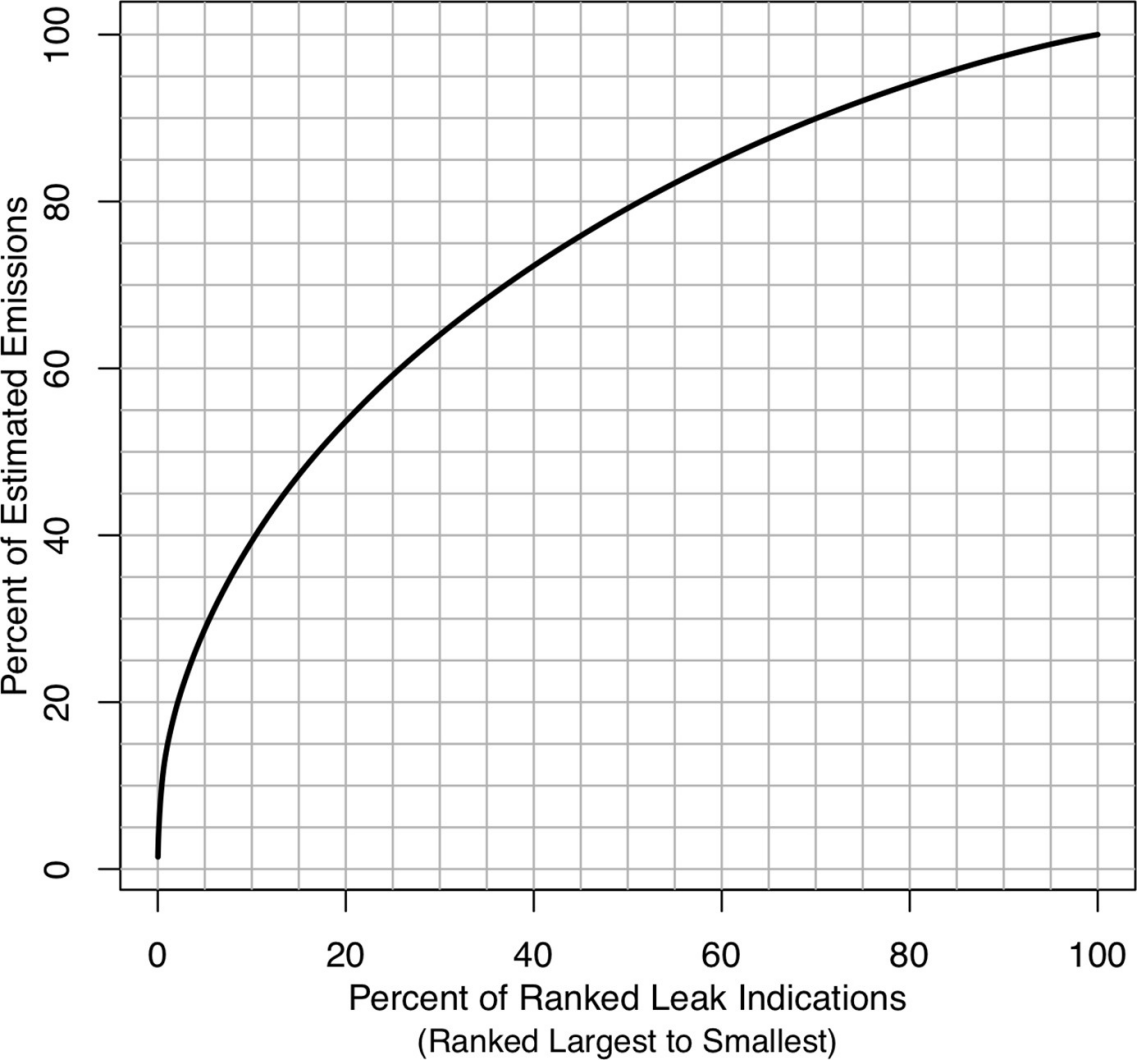
Cumulative emissions curve from the estimated sizes of 6125 leak indications. The cumulative emissions curve indicates that largest 20% of leaks account for approximately 54% of total emissions.

Fig 8 shows a cumulative emissions curve from the 6125 leak indications. Previous studies have defined the largest 5% of leaks as super-emitters [28]. In their analysis of NG leaks from 18 studies across the NG supply chain, Brandt et al. [26] propose the 5–50 rule where the largest 5% of leaks account for at least 50% of emissions. Our cumulative emissions curve indicates that the largest 5% of leak account for only 17% of emissions, suggesting that the distribution of emission rates for local NG distribution is not as heavy tailed as the distribution of leak emission rates in other segments of the NG supply chain.

It is not clear why the emission rates are not as heavy tailed as other NG sectors. It may be that very large sources are rarer, perhaps because they are readily detected by their odor and are thus rapidly found and repaired. On the other hand, there may be relatively more small leaks than in other sectors due to the numerous connections and fittings in local distribution systems. Unlike large leaks, these small leaks persist because they are either difficult to detect or do not pose a safety concern. In combination, we hypothesize that this lack of large leaks and abundance of small leaks causes a departure from the 5/50 rule observed in other studies.

### 4.2 Anomalous leaks

We developed criteria for identifying when the leak emission rate may be anomalously large and deserving the immediate attention of the LDC. Of course, any NG leak could pose a safety threat, but as leak rate increases so does the potential for gas to accumulate quickly to explosive levels. There are two cases when we flag readings as anomalously large. First, an observed peak is flagged as anomalous when its CH_4_ concentrations exceeds 20 ppm. Second, a leak indication is flagged as anomalous when its emission rate estimate (derived from multiple observations) exceeds 15 L/min. Both of these thresholds lie above the empirical 99^th^ percentile of their respective metrics. If anomalous peaks or leak indications are discovered during the survey, we report them immediately to the LDC telephone hotline.

## Conclusions, commercialization and future improvement

The sophistication, size and cost of environmental sensors has improved over the last 15 years, enabling their creative deployment on diverse mobile platforms [31–35]. These deployments generate sophisticated datasets, requiring a new generation of algorithms for extracting the relevant data products from these observations. Often, mobile sensor deployments aim to document patterns of risk exposure across diverse human populations. We support transparency and open discussion in such efforts by explicitly documenting the methods used to interpret these data.

We have presented an open source algorithm for processing data from mobile CH_4_ sources in order to identify leak indication location. The code for implementing our algorithm and an example dataset is available on GitHub. Algorithms for processing data from mobile methane surveys have been commercially produced, and we anticipate that they employ more advanced computational and analytical techniques than those presented here. Nonetheless, we have previously shown [2] that our algorithm is effective for detecting, locating, and ranking the size of natural gas leaks in urban distribution systems.

There are several ways our algorithm could be improved. First, we do not distinguish between thermogenic and biogenic CH_4_ sources, but this capability could be added by analyzing both CH_4_ and ethane concentrations. Second, we do not utilize wind data when developing our leak location estimates. The use of wind data from both the survey vehicle and local weather stations could potentially provide better estimates of leak locations, and wind data are already being used in commercially developed algorithms.

Data from mobile CH_4_ surveys are already being analyzed more broadly [22] and being used as a basis for informing repair decisions [3,4]. But we anticipate still greater potential for the development of other data products and use of data from mobile CH_4_ surveys. For example, data from mobile surveys could be coupled with existing pipeline and building infrastructure data to create a hazard map of NG infrastructure [36]. Mobile survey data products could also be developed for incorporation into decision and scheduling algorithms for prioritizing NG infrastructure and leak repairs [37].

Not every leak is found every time by mobile methane surveys. Thus, for reasons of safety and completeness, ground surveys will always remain an essential tool for managing NG distribution systems. However, as scientists, commercial software providers, and technology developers learn more about NG leaks and improve sensing technology, these algorithms will continue to be developed and updated, making mobile surveys an integral part of maintaining the safety and integrity of local distribution systems.
